# Examining Means of Reaching Adolescent Girls for Iron Supplementation in Tigray, Northern Ethiopia

**DOI:** 10.3390/nu7115449

**Published:** 2015-11-02

**Authors:** Afework Mulugeta, Masresha Tessema, Kiday H/sellasie, Omer Seid, Gebremedhin Kidane, Aweke Kebede

**Affiliations:** 1 Mekelle University, P. O. Box: 1871, Mekelle, Ethiopia; hkiday@gmail.com (K.H.); seoumer@yahoo.com (O.S.); kidanegere@yahoo.com (G.K.); 2 Ethiopian Public Health Institute, P. O. Box: 1242, Addis Ababa, Ethiopia; masresha88@gmail.com (M.T.); awekekeb@gmail.com (A.K.)

**Keywords:** adolescent girls, iron supplementation, diet diversity score, deworming

## Abstract

Background: Iron deficiency is the most prevalent nutritional deficiency in adolescent girls from the developing world. One of the recommended interventions to improve iron status in adolescent girls is iron supplementation. Yet the provision of iron supplements to adolescent girls proved to be a challenging task for the health systems across the developing world. Objective: The objective of the study was to examine means of reaching adolescent girls for iron supplementation in Northern Ethiopia. Methodology: Analytical cross-sectional study consisting of both quantitative and qualitative approaches to data collection and analysis was used in this study. Stratified multi-stage systematic random sampling technique was adopted and primary quantitative data were collected from 828 (578 school attending and 250 non school attending) adolescent girls recruited from nine districts of Tigray. The primary quantitative data were analyzed using SPSS version 20 software. The qualitative data collected through key informant interviews and focus group discussions were transcribed verbatim and qualitatively analyzed. Results: The mean (SD) age of the girls was 16.7 (1.4) years. Four hundred forty seven (54%), 355 (42.9%) and 26 (3.1%) of the adolescent girls had low, medium and high diet diversity scores, respectively. More than half, 467 (56%), of the adolescent girls believed that adolescent girls were overloaded with household jobs everyday compared to boys from their respective communities. Key informants said that, there is no adolescent nutrition message promoted in the study area. Low community awareness, perceiving iron tablet as a contraceptive, religious and cultural influences, and lack of confidence in supplementation value of iron tablets, are some of the potential barriers mentioned by the key informant and focus group discussion participants. Schools (45%), health centers (27%) and health posts (26%) were the preferred public facilities for provision of iron supplements to student adolescent girls whereas schools (11%), health centers (47%) and health posts (41%) were the preferred public facilities for provision of iron supplements to adolescent girls who were not attending schools from the study communities. Conclusion: The health posts and health centers were the preferred health facilities for iron supplementation to adolescent girls who were not attending schools while the school was the preferred facility for iron supplementation of student adolescent girls.

## 1. Introduction

Adolescence, the time period between 10 and 19 years of age, is characterized by rapid growth and development. It is a period in the human life cycle when up to 45% of skeletal growth and 15% to 25% of adult height is achieved [1] and up to 37% of total bone mass is accumulated [2]. Such remarkable physical growth and development significantly increases the macro and micro-nutrient needs of adolescents [3]. Amongst the common nutrient deficiencies commonly observed in adolescent girls is iron deficiency. Adolescent girls from the developing world are prone to iron deficiency because of the accelerated increase in the requirements for iron due to rapid pubertal growth with sharp increase in lean body mass, blood volume and red cell mass, which increases iron needs for myoglobin in muscles and hemoglobin in the blood [4]; low dietary intake of iron and poor bioavailability of iron consumed against the significant increase in requirements [5,6], high rate of infection and worm infestation [7], onset of menstruation [8], as well as the social norm of early marriage and adolescent pregnancy [9,10,11].

Recent studies from developing countries have shown that iron deficiency is still of public health significance in adolescent girls [12,13]. About 16% of student adolescent girls from Northern Ethiopia were found to be iron depleted (ferritin < 15 μg/L) and 13.7% had low iron stores (ferritin 15–30 μg/L) suggesting that iron deficiency was prevalent in this population [14]. Iron deficiency is considered to be the main cause of anemia in women, adolescents and children from developing countries. Data from 34 Demographic and Health Surveys revealed that anemia was a public health concern for adolescents and young adult females in all the 34 countries studied [15]. Various studies have reported high prevalence of anemia in adolescent girls from the developing world [6,16,17,18,19,20,21,22,23,24,25]. Similarly, between 19% and 36% of adolescent girls from Ethiopia were reported to be at risk of anemia [26,27,28,29,30].

Anemia in adolescent girls is a multi-factorial disorder that requires a multi-pronged approach for its prevention and treatment. Reduction in the prevalence of anemia among adolescent girls would not only improve maternal and newborn health but would also bring productivity gains from improved physical capacity, increased cognitive ability and intergenerational benefits [31]. The benefit-to-cost ratio of iron interventions from resource savings, improvement in cognitive development and schooling, and physical productivity was estimated to be as high as 200:1 by the 2004 Copenhagen Consensus Panel of eminent economists [32]. That is why, iron supplementation is one of the recommended interventions to reduce anemia and build iron stores in adolescent girls [33]. However, in many developing countries including Ethiopia, iron supplements are given to pregnant women who follow up antenatal care at the health facilities. Adolescents have typically been considered as low risk groups for poor iron nutrition and no longer benefiting from the iron supplementation services that usually work through the health systems. Since, adolescent girls rarely access the health system, the search for programs that go beyond the health system to reach adolescent girls is essential. Among the programs with promising results to distribute iron folic acid supplements to female students are the schools, as large numbers of girls can be reached easily where school attendance or participation is high. However, adolescent girls who are not attending schools proved to be a more challenging group of adolescents to reach for iron supplementation [34]. Thus, this study was conducted to examine means of reaching adolescent girls from Northern Ethiopia for iron supplementation.

## 2. Methodology

**Study area and study period:** The study was conducted in nine districts of Tigray, northern Ethiopia. The region is divided into northwestern and southern lowlands, 700–1500 m above sea level (masl) and the central highlands (1500–3000 masl). The region covers 54,572.6 square kms. Administratively, the region is divided into six zones and one special zone. These zones are western, northwest, central, eastern, south eastern and southern zones. The special zone is Mekelle town, which is the capital city of the region. According to the 2007 census, the population of the region were 4.3 million of which 80% were rural and 50.8% were female [35]. The study was conducted in 2013.

**Study subjects:** Adolescent girls (15–19 years) from six rural and seven urban kebelles (the smallest administrative unit in the political structure of Ethiopia) of Tigray, the northern administrative region of Ethiopia.

**Study design:** Analytical cross-sectional study that quantitatively and qualitatively explored the means of iron supplementation to adolescent girls.

**Inclusion criteria:** 15–19 years old adolescent girls were included.

**Exclusion criteria:** 15–19 years old student adolescent girls who were seriously ill, pregnant, married, had children and absent from school during the data collection period and 15–19 years old out of school adolescent girls who were seriously ill and pregnant during the data collection period were excluded.

**Sample size determination:** The minimum sample size required for the study was determined using the single population formula (*n* = Z^2^_1−α/2_ P(1 − P)/d^2^). Taking the iron folic acid supplementation coverage of 50% for maximum sample size, 95% confidence interval, 5% of margin of error, design effect of 2 and a 10% of non-response rate yielded a final sample size of 847.

**Sampling technique:** To have an effective coverage, the study was made to cover the different agro-climatic zones of the Tigray region, Northern Ethiopia. Stratified multi-stage systematic random sampling technique was adopted. One district with a high (or preparatory) school was selected by simple random sampling from each zone. When more than two schools exist in a given district, one school was selected randomly. From each selected district, two catchment kebelles of the school were selected randomly after the kebelles were stratified into rural and urban kebelles. A total of 66 adolescents (22 adolescent girls who were not students and 44 student adolescent girls) were selected from each kebelle by systematic random sampling. The land or kebelle registration book (for non student adolescents) and school roster (for student adolescent girls) were used as frames for systematically selecting the study subjects.

**Data collection:** Both quantitative and qualitative approaches to data collection and analysis were used. Primary quantitative data were collected by mainly a survey of 828 adolescent girls. Pretested, structured questionnaire consisting of several sections was used for data collection. The questionnaire was made to capture data pertaining to the socio economic and demographic characteristics, water and sanitation and nutrition. The questionnaire was developed in English and then translated into Tigrigna, the local language. However, the data extracted from the Tigrigna version of the questionnaire was translated back in to English for analysis and write up. The questionnaire was administered by trained and experienced interviewers who speak the local language. In order to supplement and triangulate the information that was gathered and analyzed quantitatively, the survey made use of qualitative approach (focus group discussion and key informant interviews) to data collection and analysis. A total of nine focus group discussions (FGDs) were conducted and in each focus group discussion, 8–10 adolescent girls were included. Individual key informant interviews were conducted with health extension and district women affairs workers, female school teachers, female students, school directors, cluster supervisors of health extension workers, health development workers, and district maternal and child health officers. Views from adolescent girls and key informants on the need and importance of addressing adolescent nutrition, iron supplementation, appropriate distribution channels of iron supplementation for adolescent girls, possible challenges in providing iron supplementation to adolescent girls and roles of the various stakeholders on improving the iron status of adolescent girls from the study communities were explored using the qualitative approach. The qualitative data were captured using field notes. These field notes were transcribed verbatim and translated into English. The moderators and field note takers during the qualitative data collection were the researchers. Before the start of the actual data collection, the facilitators of the interview and discussions were trained on the purpose of the study, contents of the questionnaire and interview guides, interviewing methods, confidentiality, interpersonal communications and other relevant issues to the study. During the training, data collectors were assessed for their interviewing skills and understanding of the contents of the questionnaire and interview guides.

**Dietary Diversity Score (DDS):** dietary diversity score was collected using the 24 h recall method and calculated as the sum of the number of different food groups consumed by the girls in the 24 h prior to the assessment. Each food group was counted only once resulting in a possible score of 0 to 9. The DDS was categorized into low dietary diversity (≤3 food groups), medium dietary diversity (4–6 food groups) and high dietary diversity (7–9 food groups) [36].

**Data management and analysis:** Data were coded and cleaned before entering into a computer. Following the coding and cleaning, data were entered into a computer for statistical analysis using SPSS version 20 (SPSS Inc., Chicago, IL, USA). Statistical analysis was combined with qualitative description and analysis. The data analysis was made to focus on univariate frequency tables and bivariate cross tabulations that identify important relationships between variables. Respondents were categorized into school going and non school going based on their current schooling status. Information that was collected through key informant (KI) interviews and FGD was transcribed, translated and categorized in themes. Finally, the data were interpreted in terms of their programmatic implications. Transcripts were compared with the detailed notes taken to verify the accuracy of the transcription.

**Ethical consideration:** Ethical clearance was obtained from the Institutional Review Board (IRB) of the College of Health Sciences of Mekelle University. Confidentiality on candidate’s information was maintained. Permission from the Regional Health Bureau and local government offices were obtained before the commencement of the study. At each of the selected study site, the local administrator in-charge was informed for consent before the commencement of the study. The purpose, general content and nature of the study were explained to each respondent to obtain verbal consent before inclusion into the study.

## 3. Results

### 3.1. Socio Demographic Characteristics

Eight hundred and forty seven participants were invited into the study and the response rate was 97.8%. Orthodox Christianity was the dominant religion of the adolescent girls from the study communities, 789 (95%). A significant proportion of adolescent girls, 250 (30%) were not attending school at the time of data collection. More than half, 467 (56%), of the adolescent girls believed that girls were overloaded with routine household activities compared to boys from their respective communities. Early marriage was amongst the harmful traditional practices reported by 13% of the adolescent girls (Table 1).

**Table 1:** Socio-demographic and economic characteristics of the adolescent girls, Tigray, Northern Ethiopia, 2013 (*n* = 828).

Characteristics		Frequency	Percent
Age in years	15	203	24.5
	16	182	22.0
	17	160	19.3
	18	181	21.9
	19	102	12.3
Subject classification	Non school going	250	30.2
	School going	578	69.8
Residence (zone)	Central	120	14.5
	Eastern	131	15.8
	Mekelle	64	7.6
	North west	131	15.9
	Southeast	126	15.2
	Southern	131	15.8
	Western	125	15.1
Religion	Christian	789	95.3
	Muslim	39	4.7
Maternal occupation	Agriculture/farmer	392	49
	Own business	123	15.4
	Wage work	45	5.6
	Household work	220	27.5
	Others	20	2.5
Paternal occupation	Agriculture /farmer	504	68.7
	Own business	115	15.7
	Wage work	95	12.9
	Others	20	2.7
% Households owning livestock	Yes	574	69.3
	No	254	30.7
Workload	Girls are overloaded than boys	467	56.4
	Boys are overloaded than girls	130	15.7
	Both are equally overloaded	231	27.9
% Believing early marriage is a harmful traditional practice	Yes	111	13.4
	No	717	86.6

Mean (Standard deviation) family size was 5.8 (1.8).

### 3.2. Health Services Provision/Utilization

The majority of the girls, 750 (91%), did not use family planning services. However, adolescent girls who utilized the services reported that they walk for an average of 52 min to get family planning services from the nearby health facilities. Treatment during illnesses, 506 (61.3%), health education, 576 (69.6%), immunization, 491 (59.5%), reproductive health services, 506 (61.3%), de-worming, 176 (21.3%) and supplementation, 274 (33.4%) were reported to be the health services provided by the nearby health facilities. The most commonly utilized health facilities for adolescent family planning services were the health centers, 71 (91%).

### 3.3. Water and Sanitary Conditions

The average time spent to fetch water for domestic consumption was reported to be 30 min. Burial or burning and collection by private establishments were reported to be the preferred methods of disposing waste in 46% and 37% of the households, respectively. Dumping on a street, nearby river or open space was practiced by 7.4% of the households. The majority of the households (90%) had an improved water source (tap water, hand pumps, protected springs or protected wells) and about one third (34%) of the households of the adolescent girls walk for more than 30 min to get water for domestic use. However, large proportions (84%) of the households do not treat their water to make it safer. Improved latrine (flush toilet or pit latrine with floor/slab) was available in 82% of the adolescent girls’ households. Soap was the most common sanitizing agent used for hand washing after using a toilet by the majority (65%) of the households.

### 3.4. Dietary Diversity Score (DDS)

The diet diversity score was calculated by simply summing the number of different food groups consumed within 24 h prior to the date of data collection. Six hundred and one (72.6%), 346 (41.8%), 264 (31.9%) and 134 (16.2%) of the adolescent girls reported that they consumed legumes, dark green leafy vegetables, other vitamin A rich fruits and vegetables and other fruits and vegetables, respectively with in 24 h prior to the survey. The consumption of meat, poultry and fish, which are good sources of bio-available iron, was also low. The mean DDS of the adolescent girls was found to be 3.5. About 447 (54%), 355 (42.9%) and 26 (3.1%) of the adolescent girls had low, medium and high diet diversity scores, respectively (Table 2).

**Table 2:** Food groups consumed by the adolescent girls 24 h prior to the survey in Tigray, Northern Ethiopia, 2013 (*n* = 828).

Food Groups		*n*	Percent
Starchy staples (cereals such as maize, rice, wheat, barley, sorghum, millet, teff or any other grains or foods made from these (e.g., bread, injera, roasted cereals, porridge, spaghetti, macaroni, *etc.*) and white roots and tubers such as white potatoes, or other foods made from roots)	Yes	827	99.9
Dark green leafy vegetables (such as kale, spinach)	Yes	346	41.8
Other vitamin A rich fruits and vegetables (vitamin A rich vegetables and tubers such as pumpkin, carrot, squash, orange flesh sweet potato, *etc.*; vitamin A rich fruits such as mango, papaya, peach, *etc.*; and red palm oil)	Yes	264	31.9
Other fruits and vegetables (vegetables such as tomato, onion, egg plant, *etc.* and other fruits)	Yes	134	16.2
Organ meat (liver, kidney, heart, blood based foods, *etc.*)	Yes	21	2.5
Meat and fish (flesh meats such as beef, pork, lamb, goat, sheep, chicken and fish and sea food)	Yes	268	32.4
Eggs	Yes	232	28.2
Legume, nuts and seeds (beans, peas, lentils, nuts, peanut butter, *etc.*)	Yes	601	72.6
Milk and milk products (milk, cheese, yoghurt)	Yes	219	26.5
Mean diet diversity score	3.5		

### 3.5. Nutrition

Slightly above a third, 304 (37%), of the adolescent girls were aware that iron deficiency anemia could be prevented by iron supplementation through accessible facilities especially health systems and schools. Large proportion, 682 (82%) of the adolescent girls perceived that they were not anemic at the time of data collection. Fatigue (42%) and listlessness (36%) were the most commonly reported symptoms of anaemia. The consumption of iron rich foods was indicated as one of the best remedies for anaemia in adolescent girls. The low intake of fruits and vegetables and dairy products were amongst the poor dietary practices of adolescent girls from the study communities. Significant proportion of the adolescent girls (96%) believed that adolescent girls need to be targeted for nutrition. Schools (45%), health centers (27%) and health posts (26%) were the preferred public facilities for provision of iron supplements to school going adolescent girls. Similarly, schools (11%), health centers (47%) and health posts (41%) were the preferred public facilities for provision of iron supplements to non school going adolescent girls from the study communities (Table 3).

### 3.6. Qualitative Study Findings

The adolescent girls expressed their perceptions regarding the roles of the various stakeholders on improving the iron status of adolescent girls from the study communities. According to the focus group discussants, the various stakeholders at different levels need to take account of the current status of iron deficiency anemia in adolescent girls and do their level best to improve iron nutrition through the involvement of the various actors at different levels. The adolescent girls identified schools, health facilities, local government offices, community leaders and peers as stakeholders to improve nutritional status of adolescent girls and facilitate the provision of iron supplementation in their respective communities (Table 4).

**Table 3:** Knowledge regarding nutrition, anemia and iron supplementation and current dietary practices of adolescent girls from Tigray, Northern Ethiopia, 2013 (*n* = 828).

Variable		Frequency	Percent
Adolescence is a critical period for nutrition	Yes	777	94.0
	No	51	6.0
Are you aware of the fact that there is a good relationship between health and nutrition?	Yes	791	95.6
	No	37	4.4
Where did you get information or advices regarding good nutrition?	Health facility	352	44.6
	Schools	587	74.3
	Friends	122	14.7
	Health development army	58	7.4
Is deworming for intestinal parasites given to adolescent girls from this community?	Yes	163	19.7
	No	665	80.3
Dieting practices by adolescent girls in the study communities	Snacking, usually on energy-dense foods	128	15.5
	Meal skipping, particularly breakfast, or irregular meals	202	24.5
	Wide use of fast foods	43	5.2
	Low consumption of fruits and vegetables	531	64.3
	Low consumption of dairy products	499	60.4
Should adolescent girls be targeted for nutrition	Yes	748	95.5
	No	80	4.5
Are messages on adolescent nutrition promoted in the community?	Yes	219	27.0
	No	609	73.0
The best media to disseminate information about adolescent nutrition	Radio	670	83.6
	TV	67	12.1
	Fm radio	7	0.9
	Others, please specify:	27	3.4
Mention symptoms of anemia	Listlessness (lacking energy)	300	36.2
	Heart palpitations upon exertion	81	9.8
	Fatigue, irritability	349	42.1
	Paleness of skin	148	17.9
	Cracking of lips and tongue	36	4.3
	General feeling of poor health	114	13.8
	Poor physical growth in children	5	0.6
	Poor work out put in adults	44	5.3
Prevention of anemia	Eating iron rich foods	725	87.6
	Taking iron supplements	304	36.7
	Do not know	68	8.2
Perceived iron status	Normal	682	82.4
	Anemic	146	17.6
Preferred facility for iron supplementation to school going adolescent girls	Health post	167	26.3
	School	287	45.1
	Health centre	170	26.7
	Hospital	12	1.9
Preferred facility for iron supplementation to non school going adolescent girls	Health post	102	40.5
	School	28	11.1
	Health centre	118	46.8
	Hospital	4	1.6

**Table 4:** Responsibilities of the various actors in facilitating iron supplementation to adolescent girls from Northern Ethiopia.

Facilities	Key Informants	Focus Group Discussions
Schools	Provision of nutrition education on anemia and iron supplementation, establishment of clinics in schools, inclusion of nutrition sessions in the classes, preparation of educational materials, establishment of nutrition clubs and presentations in the mini media	Nutrition education to clear the misconception about iron supplements, provision of iron tablets to adolescent girls and create favourable environment for iron supplementation for female students.
Family members	Encourage adolescent girls to take supplements and allow them to take iron supplements either from schools or health facilities	Encourage intake of iron supplements
Local authorities	Awareness creation, community mobilization, make sure that it is included in the plan of action and work plans and monitoring and evaluation of the implementation of the intervention	Educate and mobilize the community
Community	Encourage and convince their children to use it freely and encourage the agenda of adolescent nutrition in the community conversation and other relevant forums such as churches and mosques	Encourage iron supplementation to be freely practiced in the community
Peers	Identify role models for the prevention of anemia and iron supplementation	Discuss anemia in adolescents and iron supplementation in the 1 to 5 networking, a type of local organization that will enable all network members to implement health services such as iron folic acid supplementation to adolescent girls effectively and efficiently.
Community influentials	Educate the community and include anemia and iron supplementation in community discussions	Educate the community and include anemia and iron supplementation in community discussions

Key informants recommended that adolescent nutrition must be incorporated into the school curriculum and call for the establishment of school clinics and girl’s nutrition clubs and the inclusion of debates about adolescent nutrition and the importance of iron supplementation to adolescent girls in biology and other relevant courses. The adolescents also indicated that the support from home and family members had an influence on their compliance to iron supplementation. Moreover, the adolescent girls have indicated that the local government offices and community leaders could have a role to play in supporting the iron supplementation efforts to improve the iron status of adolescent girls.

The adolescent girls reported that lack of awareness; wrong perception about iron tablets (viewed as contraceptive which leads to infertility); perceived feeling of illness due to the association between tablets and illness; intake of tablets without diagnosis for deficiency of iron and doubt in the interventions were the potential barriers for iron supplementation in adolescent girls from the study communities. Among the barriers identified from the key informant interviews and focus group discussions were low community awareness, scepticism in the intervention, rejection of intake by the family, perceptions associated with the tablet as a cause of infertility, lack of courage to take tablets while healthy, beliefs that iron tablets increase bleeding during menstruation, side effects, association of taking supplements for an extended period with Anti Retroviral Therapy (ART) regimen and humiliation from male students for taking iron supplements.

On the other hand, the potential facilitators for the acceptability of iron supplementation to adolescents were provision of adequate counseling and training to adolescents and families, women association, women league, religious leaders and women development armies; diagnostic testing and provision of iron tablets for anemic adolescents; adequate supply of iron tablets in the nearby health facilities and schools and training of teachers especially female teachers on the importance of iron nutrition, consequences of iron deficiency and iron supplementation.

The well organized bottom up (kebelle to district) political structure; the easy accessibility and availability of schools and health facilities; availability of professionals such as teachers and health extension workers (HEWs); the organization of women in various institutions such as women development armies, the women league, women affairs group, women association and youth association; access to media especially radio and TV and increased school enrollment rate of girls were mentioned as the potential facilitators of the implementation of iron supplementation at various levels.

Death, fatigue and tiredness were mentioned as the consequences of anemia by the adolescent girls included in the study. Amongst the fading away perceptions that used to influence priority behaviors that affect the nutritional status of adolescent girls were heavy work load, food taboos like the avoidance of egg (believed to block sexual organs of girls), animal products (enhance growth and early maturation), ground nut (increases sexual interest) and hot drinks such as tea (believed to enhance growth and increase blood loss during menstruation in girls) and gender preferences where males are given priority as males are believed to be more susceptible to malnutrition.

## 4. Discussion

Schools (45%) were the preferred public facilities for provision of iron supplements to school going adolescent girls. Different studies have shown school-based iron and folic acid supplementation to be a feasible and effective intervention to prevent a decline in hemoglobin and reduce the prevalence of iron deficiency anemia in adolescent girls. A study from the Philippines reported that weekly iron supplements given by teachers had sustained the hemoglobin concentration of schoolchildren and school teachers were able to give children the iron tablets with a high level of compliance [37]. School teachers from Mali gave weekly iron tablets to their students for 10 weeks and achieved 90% coverage that resulted in a 17% reduction in anemia prevalence [38]. School based studies from Bolivia [39] and Malaysia [40] have shown similar benefits of iron supplements for children. Another study from Peru have shown that iron supplementation through the school system were efficacious in preventing iron deficiency, increasing hemoglobin concentrations and reducing anemia in adolescent girls [41]. Alternatively, the health facilities specifically the health centers (47%) and health posts (41%) were the preferred public facilities for provision of iron supplements to non school going adolescent girls from the study communities. Studies have indicated that health facilities were also efficient facilities to reach adolescent girls for iron supplementation [42]. This evidence suggests that the use of schools and health facilities (health centers and health posts) as the administration channels could serve as the most feasible and effective means of reaching school going and non school going adolescent girls for iron supplementation from our study communities, respectively.

Although iron supplementation will increase hemoglobin concentration, increase iron stores and reduce anemia in adolescent girls, the positive effect on iron status will be temporary if the diets do not contain adequate bioavailable iron. Dietary diversity which is the number of foods groups over a reference period commonly in the 24 h prior to the data collection period is widely recognized as a key dimension of diet quality in individuals and households. Diet diversity is strongly associated with nutrient adequacy [43]. Our results showed that the mean DDS was 3.5. About 54%, 43% and only 3% of the adolescent girls had low, medium and high diet diversity scores, respectively suggesting that low quality monotonous diets were the norm for adolescent girls from the study communities. Their diets were dominated by cereal based staple foods but lacked vegetables, fruits and animal-source foods aggravating the risks for a range of micronutrient deficiencies including iron. Fruits aren’t good sources of iron. They are, however, a good source of ascorbic acid which facilitates absorption of nonheme iron. Fruits, vegetables and animal source foods are expensive and that is why the consumption of these foods was poor by the adolescent girls from the study communities. This will help explain why supplementation is an option to address iron deficiency in adolescent girls from the study communities. Our results suggested that there is a good case for iron supplementation to adolescent girls through the schools and health facilities as the intake of foods with adequate bioavailable iron was minimal in the adolescent girls from the study communities.

Schools were identified as the best places to provide counseling and education on the need for iron supplementation by the adolescent girls. Thus, sustained counseling and education should be given about iron and its advantages for adolescent girls in the schools and the schools should take the responsibility to provide iron supplements to their students on regular intervals. Similarly, the schools must serve as platforms for health professionals and teachers to discuss nutrition and health issues of adolescent girls. Schools should educate their students to clarify misinformation about iron supplements. There are perceptions within the community that iron supplements are taken not to correct iron deficiency but as contraceptive pills. The incorporation of adolescent nutrition into the school curriculum would require decent knowledge on nutrition from the teachers and the health sector in collaboration with the education sector should organize trainings to teachers on such critical and essential topics. Moreover, the nutrition issues of adolescents can be discussed in the mini media and essential messages communicated to students prior to the singing of National Anthem in the morning.

Health facilities were also identified as another potential platform for iron supplementation to adolescent girls during the qualitative data collection. The health facilities could supply iron tablets, follow up the implementation of iron supplementation and provide health education on iron nutrition and the consequences of iron deficiency to adolescent girls. Moreover, health facilities could support the iron supplementation efforts through diagnosis of iron deficiency when adolescents visit health facilities and lead the iron supplementation efforts and provision of health education on the safety and possible side effects of the intervention.

The various actors especially local government offices, family members and community leaders could play a role in supporting the iron supplementation efforts to adolescent girls. Consistent encouragement and follow up of family members would ensure compliance with iron supplementation by adolescent girls. Education and counseling need to be given to the immediate family members on the need for iron supplementation for adolescent girls. Good parental knowledge about iron nutrition and its consequences would encourage girls to use iron tablets. The local government offices can easily make iron nutrition and supplementation as a timely and topical issue in their respective localities for community mobilization, awareness creation at the community level and ensuring the full participation of the women development armies to supporting the iron supplementation to adolescent girls. Involvement of community and religious leaders can also influence the community through various approaches such as churches and mosques. Peers were also mentioned as influential concerning iron supplementation use in adolescent girls. Easy adopters can greatly influence others for iron supplementation in adolescent girls who attend, and girls who do not attend, school, suggesting that peers could serve as motivators, supporters and educators for improved behavior towards iron supplementation during adolescence.

The use of mixed approaches of data collection (quantitative and qualitative), random selection of the communities and participants and use of large sample size were the strengths of this study. The findings from the qualitative study have provided a deep understanding for the potential barriers and facilitators of iron supplementation in both the adolescent girls who were going to school and the girls who were not going to school that would not be achieved through quantitative studies. One of the limitations of the study could be the voluntary participation of the girls as it might have made room for exclusion of the experiences of those who did not wish to participate in the study for any reason.

## 5. Conclusions

In conclusion, adolescence provides an opportunity to prepare for a healthy reproductive life. Through iron supplementation during adolescence, the adolescent girls can enter pregnancy without iron nutritional problems originated in the past and with no serious iron deficiency handicaps. Considering the quite long lasting effects of iron deficiency and the biological and operational feasibility and the effectiveness of the intervention, correction of iron deficiency in adolescent girls through iron supplementation through appropriate channels is critical. In this study, the health posts and health centers were identified as the best distribution channels for iron supplementation to adolescent girls who were not attending school while the school systems were identified to be the preferred means of reaching school adolescent girls for iron supplementation.

## References

[1] Rees J.M., Christine M.T., Adams G. (1989). Nutritional influences on physical growth and behavior in adolescence. Biology of Adolescent Behaviour and Development.

[2] Key J.D., Key L.L. (1994). Calcium needs of adolescents. Curr. Opin. Pediatr..

[3] Lifshitz F., Tarim O., Smith M.M. (1993). Nutrition in adolescence. Endocr. Metab. Clinics. N. Am..

[4] Beard J.L. (2000). Iron requirements in adolescent females. J. Nutr..

[5] Allen L. (2000). Prevalence and causes of nutritional anaemia. Nutritional Anaemia.

[6] Creed-Kanashiro H.M., Uribe T.G., Bartolini R.M., Fukumoto M.N., Lopez T.T., Zavaleta N.M., Bentley M.E. (2000). Improving Dietary Intake to Prevent Anemia in Adolescent Girls through Community Kitchens in a Periurban Population of Lima. Peru. J. Nutr..

[7] Hershko C. (1993). Iron, infection and immune function. Proc. Nutr. Soc..

[8] Hallberg L., Rossander-Hulthen L. (1991). Iron requirements in menstruating women. Am. J. Clin. Nutr..

[9] Kotadawala P.J., Bhave S.Y., Nair M.K.C. (2000). Gynecological problems in adolescent girls. IAP Manual on Adolescent Care.

[10] Sharma A.K., Verma K., Khatri S., Kannan A.T. (2001). Pregnancy in adolescents: A study of risks and outcome in Eastern Nepal. Ind. Paediatr..

[11] Sharma V., Sharma A. (1992). Health profile of pregnant adolescents among selected tribal population of Rajasthan. Ind. J. Adolesc. Health.

[12] De Andrade Cairo R.C., Rodrigues Silva L., Nadya Carneiro Bustani N., Ferreira Marques C.D. (2014). Iron deficiency anemia in adolescents; a literature review. Nutr. Hosp..

[13] Drorbaugh N., Neumann C.G. (2009). Micronutrient deficiencies in food aid beneficiaries: A review of seven African countries. Afr. J. Food Agric. Nutr. Dev..

[14] Mulugeta A., Gebre M., Abdelkadir M., Gebretsadik A., Gebreyesus A., Stoecker B.J. (2010). Iron deficiency in adolescent school girls from Tigray, Northern Ethiopia. FASEB J..

[15] Yasutake S., He H., Decker M.R., Sonenstein F.L., Astone N.M. (2013). Anemia among Adolescent and Young Women in Low-and-Middle-Income Countries. Int. J. Child Health Nutr..

[16] Deshpande N.S., Karva D., Agarkhedkar S., Deshpande S. (2013). Prevalence of anemia in adolescent girls and its co-relation with demographic factors. Int. J. Med. Public Health.

[17] Biradar S.S., Biradar S.P., Alatagi A.C., Wantamutte A.S., Malur P.R. (2012). Prevalence of Anemia among Adolescent Girls: A One Year Cross-Sectional Study. J. Clin. Diagn. Res..

[18] Siddharam S.M., Venketesh G.M., Thejeshwari H.L. (2011). A Study of Anemia Among Adolescent Girls in Rural Area of Hassan district, Karnataka, South India. Int. J. Biol. Med. Res..

[19] Kakkar R., Kakkar M., Kandpal S.D., Jethani S. (2011). Study of anemia in adolescent school girls of Bhopal. Indian J. Community Health.

[20] Pattnaik S., Patnaik L., Kumar A., Sahu T. (2013). Prevalence of Anemia among adolescent girls in a rural area of Odisha and its epidemiological correlates. Indian J. Mater. Child Health.

[21] Miah M.S., Rahman M.N., Prodhan U.K., Linkon M.R., Madumita, Rahman M.S. (2014). Prevalence of iron deficiency anemia among adolescent girls and its risk factors in Tangail region of Bangladesh. Int. J. Res. Eng. Technol..

[22] De la Cruz-Góngora V., Gaona B., Villalpando S., Shamah-Levy T., Robledo R. (2006). Anemia and iron, zinc, copper and magnesium deficiency in Mexican adolescents. Natl. Health Nutr. Survey.

[23] Hettiarachchi M., Liyanage C., Wickremasinghe R., Hilmers D.C., Abrams S. (2006). Prevalence and severity of micronutrient deficiency: A cross-sectional study among adolescents in Sri Lanka. Asia Pac. J. Clin. Nutr..

[24] Bamidele J.O., Abodunrin O.L., Olajide F.O., Oke Y.F. (2010). Prevalence and determinants of anemia among primary school pupils of a Peri-urban community in Osun State, Nigeria. Int. J. Adolesc. Med. Health.

[25] Sinha A.K., Singh Karki G.M., Karna K.K. (2012). Prevalence of Anemia amongst Adolescents in Biratnagar, Morang District. Nepal. Int. J. Pharm. Biol. Arch..

[26] Desalegn A., Mossie A., Gedefaw L. (2014). Nutritional iron deficiency anemia: Magnitude and Its Predictors among School Age Children, Southwest Ethiopia: A Community Based Cross-Sectional Study. PLoS ONE.

[27] Assefa A., Mossie A., Hamza L. (2014). Prevalence and severity of anemia among school children in Jimma Town, Southwest Ethiopia. BMC Hematol..

[28] Mesfin F., Berhane Y., Worku A. (2015). Anemia among Primary School Children in Eastern Ethiopia. PLoS ONE.

[29] Gutema B., Adiss W., Asress Y., Gedefaw L. (2014). Anemia and associated factors among school-age children in Filtu Town, Somali region, Southeast Ethiopia. BMC Hematol..

[30] Seid O.A., Tadesse K., Gebremedhin A. (2015). Iron deficiency anemia is moderate public health problem among school going adolescent girls in Berahle district, Afar, northeast Ethiopia. J. Food Nutr. Sci..

[31] Alderman H., Horton S., Kraemer K., Zimmermann M.B. (2007). The economics of addressing nutritional anemia. Nutritional Anemia.

[32] World Health Organization (WHO) (2011). Prevention of iron deficiency anemia in adolescents. Role of Weekly Iron and Folic Acid Supplementation.

[33] Behrmann J.R., Alderman H., Hoddinott J. (2004). Hunger and malnutrition. Copenhagen Consensus Challenge Paper.

[34] Joshi M., Gumashta R. (2013). Weekly Iron Folate Supplementation in Adolescent Girls–An Effective Nutritional Measure for the Management of Iron Deficiency Anaemia. Glob. J. Health Sci..

[35] Bulliy G., Mallick G., Seth G.S., Kar S.K. (2007). Hemoglobin status of non school going girls in 3 districts of Orissa, India. Int. J. Adolesc. Med. Health.

[36] Central Statistical Agency (CSA) (2010). The 2007 Population and Housing Census of Ethiopia.

[37] Swindale A., Bilinsky P. (2006). Household Dietary Diversity Score (HDDS) for Measurement of Household Food Access: Indicator Guide Version 2.

[38] Roschnik N., Parawan A., Melba Andrea B., Teresita C., Andrew H. (2004). Weekly iron supplements given by teachers sustain the hemoglobin concentration of schoolchildren in the Philippines. Trop. Med. Int. Health.

[39] Hall A., Roschnik N., Ouattara F., Touré I., Maiga F., Sacko M., Moestue H., Bendech M.A. (2002). A randomized trial in Mali of the effectiveness of weekly iron supplements given by teachers on the hemoglobin concentrations of schoolchildren. Public Health Nutr..

[40] Aguayo V.M. (2000). School-administered weekly iron supplementation–effect on the growth and hemoglobin status of non-anemic Bolivian school-age children. A randomized placebo-controlled trial. Eur. J. Nutr..

[41] Tee E.S., Kandiah M., Awin N., Chong S.M., Satgunasingam N., Kamarudin L., Milani S., Dugdale A.E., Viteri F.E. (1999). School-administered weekly iron-folate supplements improve hemoglobin and ferritin concentrations in Malaysian adolescent girls. Am. J. Clin. Nutr..

[42] Zavaleta N., Respicio G., Garcia T. (2000). Efficacy and Acceptability of Two Iron Supplementation Schedules in Adolescent School Girls in Lima. Peru. J. Nutr..

[43] Foote J., Murphy S., Wilkens L., Basiotis P., Carlson A. (2004). Dietary variety increases the probability of nutrient adequacy among adults. J. Nutr..

